# Impaired binocular vision with bangerter foil and its influence on fine motor skills: a clinical observational, randomized and cross-sectional study

**DOI:** 10.1007/s00417-026-07150-2

**Published:** 2026-03-23

**Authors:** Pilar Granados-Delgado, Miriam Casares-López, Francesco Martino, Rosario González Anera, José Juan Castro-Torres

**Affiliations:** Department of Optics/Laboratory of Vision Sciences and Applications, Faculty of Sciences, University of Granada, Granada, Spain

**Keywords:** Binocular vision, Fine motor skills, Visual impairment, Bangerter foils, Stereoacuity, Contrast sensitivity, Disability glare, Visual acuity

## Abstract

**Purpose:**

To analyze the influence of simulated impaired binocular vision on visual performance and fine motor skills (FMS).

**Methods:**

A total of 33 young and healthy participants were included. A Bangerter foil 0.8 was employed to induce impaired binocular vision. Visual performance was assessed randomized, under three binocular viewing conditions: binocular baseline, wearing a Bangerter foil on dominant eye, and wearing Bangerter foils on both eyes, by visual functions: visual acuity, contrast sensitivity, disability glare, and stereopsis. Five tests evaluated fine motor skills: Purdue, O´Connor, and Grooved Pegboard; water pouring and threading task. The Overall Visual Performance Score (OVPS) and Overall Fine Motor Skills Score (OFMSS) were calculated.

**Results:**

Binocular visual performance was significantly worse when deterioration was induced simultaneously on both eyes for all visual functions tested (*p* < 0.05). There were no significant differences in visual acuity and near contrast sensitivity between deteriorated conditions in only one eye and baseline. However, the two visual impairment conditions differed significantly, except for stereoacuity. For FMS, the deterioration factor was also significant (*p* < 0.05). In terms of OVPS and OFMSS, the greater the induced binocular dysfunction, the worse the visual performance and ability to perform fine motor tasks (rho = 0.334; *p* < 0.001).

**Conclusions:**

These findings suggest that mild monocular or binocular induced impairments have an impact on the visual functions measured and also have a marked effect on the performance of manual tasks that require fine motor skills. These results may be comparable to those from patients with recent-onset edema or cataracts; nevertheless, a full reproduction of the optical and neural effects of genuine pathology by the simulations is not achieved.

**Supplementary Information:**

The online version contains supplementary material available at 10.1007/s00417-026-07150-2.

## Key Message

**What is known:**

- Bangerter foils are commonly used to simulate visual deterioration and have been shown to reduce visual acuity, contrast sensitivity, and stereoacuity.
- Bangerter foils (0.8) induce a mild visual impairment that may be analogous to that caused by incipient cataracts or corneal edema.

**What is new:**

- Induced mild visual impairment, both monocular and binocular, significantly deteriorated visual performance (VA, CS, disability glare, stereopsis) and manual dexterity.
- Fine motor tasks, especially daily-life tasks such as needle threading and water pouring, are more sensitive to visual impairment than standardized pegboard-based tests.
- A significant correlation was found between overall visual performance and fine motor skills, suggesting that mild visual deterioration comparable to early-stage cataracts or corneal dystrophies can have an impact on daily tasks involving fine motor skills.

**Supplementary Information:**

The online version contains supplementary material available at 10.1007/s00417-026-07150-2.

## Introduction

In recent years, different studies have shown interest in studying the influence of vision on daily activities such as driving, reading, and other visuomotor tasks [1–6], including those involving fine motor skills [5, 7–9], because it entails social and economic costs [8, 10]. Therefore, understanding visual impairment in these daily tasks is vital to improve their quality of life. However, this is a complex issue that involves different aspects (hand movements and the visual system). The assessment of binocular visual performance could represent a key aspect of this comprehensive relationship [11, 12]. Binocular vision allows us to see depth based on the horizontal binocular disparity [13]. It involves other visual functions as binocular visual acuity (VA), contrast sensitivity (CS), and disability glare (DG) [14]. The visual impairment (either monocular or binocular) also negatively affects binocular summation. In addition, monocular visual deterioration leads to interocular differences (ID), as shown by previous studies [5, 15–17].

However, the deterioration observed in binocular vision and, therefore, its impact on the performance of daily tasks, may depend on the degree of impairment. There are different levels of visual impairment caused by ocular pathologies as cataracts, corneal dystrophies or maculopathies (diseases in the central retina), and visual dysfunctions as amblyopia (a unilateral or, less often, bilateral [18] or strabismus). Depending on the degree of these ocular pathologies or conditions, VA may be reduced [7]. In the specific case of cataracts and pathologies that cause a decrease in corneal or lens transparency, there is also an increase in straylight, causing alterations in contrast sensitivity [19]. The effect of monocular degraded vision or the deterioration of stereoacuity on sensorimotor performance has been studied previously, showing the correlation between the impairment in these functions and a grasping task [20, 21].

Researchers simulate visual impairment in people with normal vision using Bangerter foils or plus lenses to characterize and control the impairment [22]. Bangerter foils demonstrated a significant reduction in accuracy when performing a water pouring task with bilateral impairment [5]. No changes were observed in saccades, reaching movements, and eye-hand coordination during a simple reach-to-touch task using lenses [23]. However, in the case of deteriorated stereopsis, grasp performance was disrupted [24].

In particular, the Bangerter foils block light transmission and produces different levels of deterioration on the retinal-image quality and causes deterioration of visual functions such as VA, CS, stereoacuity, and DG [25–27]. These foils are primarily used for the treatment of amblyopia [28], but can also be used for the simulation of different levels of visual deterioration (as cataracts or corneal disorders).

Despite these findings, to date, very little is known about how a mild, yet common visual impairment affects visual quality and binocular function and its impact on the performance of daily manual tasks. Additionally, although previous studies have used Bangerter foils to simulate mild visual impairment, further research is required into the possible implications across different visual functions and tests (particularly stereopsis), and its impact on fine motor skills used in everyday life. These less compromising viewing conditions are found in incipient unilateral and bilateral cataracts, as well as various types of corneal dystrophies. Thus, this study aimed to investigate the effects of induced monocular and binocular mild visual deterioration with Bangerter foils on VA, CS, DG, and stereoacuity, and the influence on eye-hand coordination in visually normal participants.

## Methods

### Subjects

**Fig. 1:**
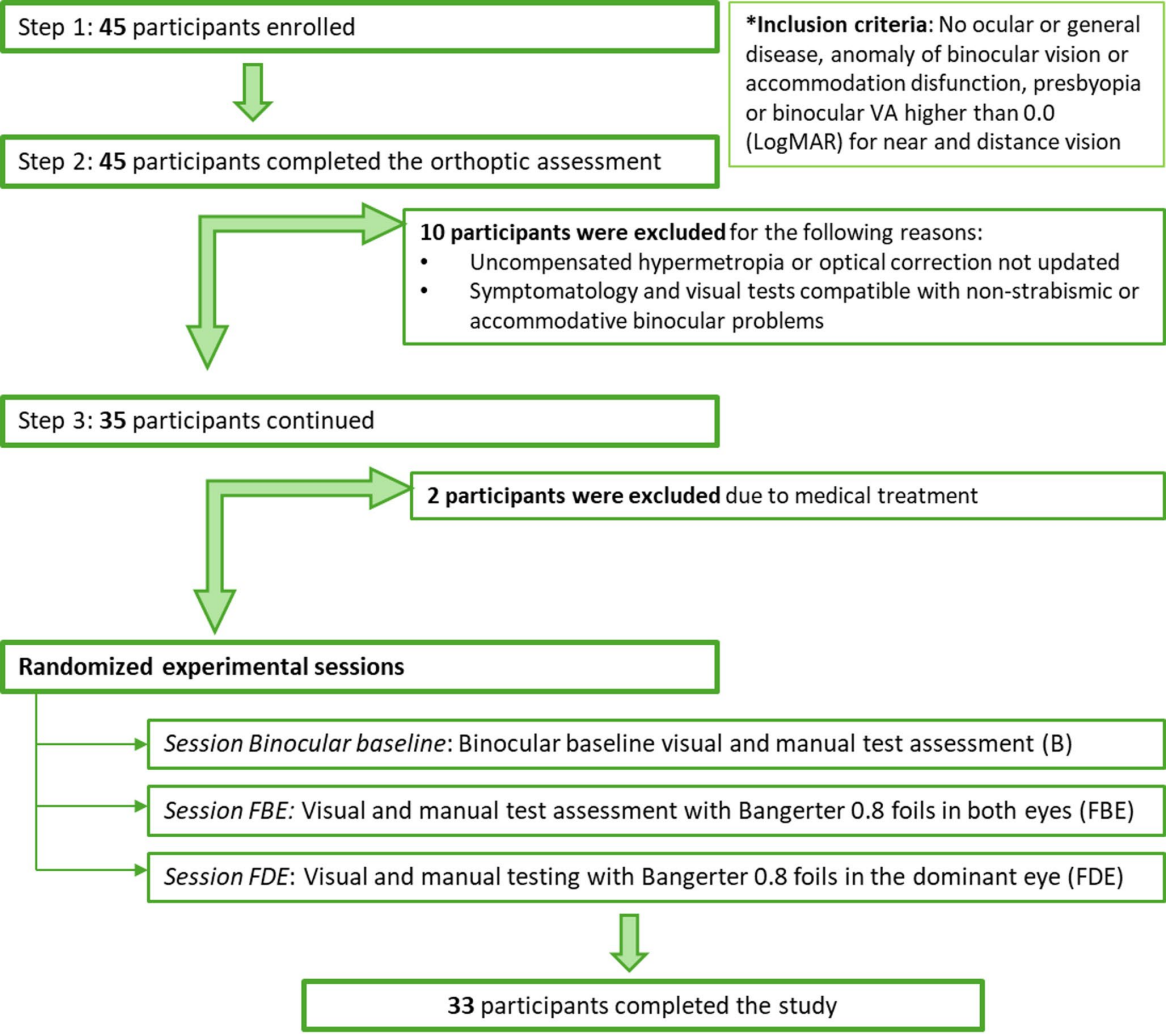
The flow diagram shows the inclusion criteria, recruitment process, and the procedure followed by participants who completed the study

A total of 33 participants were included in the study (20 females and 13 males, mean age 25.5 ± 4.9 years) after excluding 12 out of a total of 45 subjects. The inclusion criteria and recruitment process are summarized in Fig. 1. All participants signed a written informed consent form in accordance with the Helsinki Declaration (World Medical Association, 2009). The procedures described were approved by the Human Research Ethics Committee of the University of Granada (1256/CEIH/2020).

### Foil to induce visual deterioration

To induce a deterioration in binocular vision, we used a Bangerter foil (Ryser Optik, St Gallen, Switzerland) of grade 0.8 (BF_0.8). These foils produce image distortions affecting visual functions such as VA and CS [15, 22, 27]. The expected visual acuity by the manufacturer, using this foil, is 0.1 logMAR.

The average integrated transmittance obtained for the pair of Bangerter foils used in our study was 0.778 ± 0.076. They were measured with a SpectraScan PR-745 spectroradiometer (Photo Research, Inc. CA, USA) [29]. The same pair of Bangerter foils (right eye and left eye) was used for all participants.

### Procedures

Before performing the visual and manual dexterity tests, the optical correction of the participants was checked and adjusted. This assertion was subsequently corroborated. As demonstrated in Fig. 1, all participants included met the stipulated inclusion criteria. The mean sphere value was − 2.28 ± 2.57 D, and the cylinder value was − 0.74 ± 0.52 D. The mean anisometropia was lower than 1.75 D (mean 0.75 ± 0.43 D). Participants underwent three randomized experimental sessions separated in time, one week under binocular viewing conditions: baseline, i.e. with no foil (BB), wearing a Bangerter foil on the dominant eye (FDE), and wearing Bangerter foils on both eyes (FBE) (Fig. 1). The sensorial ocular dominance was assessed using R + L test from Randot Stereotest (Stereo Optical C.O. 2009). The BF_0.8 were placed on the front side of ophthalmic lenses with no optical power and mounted in the same optical frame for all participants.

The same pair of Bangerter foils was used for all participants. Lenses were cleaned before and after each session with a chamois cloth, without alcohol or chemical agents, and stored in a dedicated protective case to avoid dust and light exposure. Foil stability was verified by measuring its processing speed after completion of data collection; this value is reported in the manuscript and indicates no relevant degradation.

### Visual performance

In addition to randomizing the experimental sessions, the performance of the visual and motor skills tasks described below was also randomized within each experimental session. This yielded 5! = 120 possible combinations when only the 5 manual tasks were considered, and 6! = 720 combinations when the 6 visual tests are taken into account. Considering randomization across three sessions, manual tasks, and visual tasks, millions of possible execution sequences would be identified. This ensured that the execution pattern would be unique for each participant, thus minimizing systematic order-related bias.

Visual performance was binocularly assessed under three experimental sessions (BB, FDE and FBE) by means of different visual functions, including visual acuity (VA), contrast sensitivity (CS), disability glare (DG), and stereopsis.

Near (0.5 m) and distance (5.5 m) VA was assessed using OptoTab visual acuity screening test (SmarThings4Vision, Zaragoza, Spain) [9, 30]. CS was tested at 0.5 m using a sinusoidal grid test (SmarThings4Vision, Zaragoza, Spain). Five spatial frequencies were assessed: 1.5, 3.0, 6.0, 12.0, and 18.0 cycles per degree (cpd), and nine contrast levels were evaluated for each spatial frequency and was reported as the averaged CS for all spatial frequencies analyzed [9]. DG was obtained as the difference between the contrast threshold with glare and without glare. The CGT-1000 glare tester (Takagi Seiko Co, Naganoken, Japan) was used [9]. To assess the capacity to perceive depth (stereopsis), three different tests were employed: Frisby, Randot Circles Test of the Randot Stereotest (performed at 40 cm), and Polarized OptoTab stereotest (performed at 5.5 m) [16, 21, 24, 31]. Both Randot and Optotab are polarized tests; in contrast, Frisby stereotest (Stereotest Ltd., Sheffield, UK) shows real depth [31].

To obtain a single metric for the visual performance including all visual functions assessed, the Overall Visual Performance Score (OVPS) was calculated, a parameter used in previous works [9, 32]. Positives z-scores represented a better performance than the mean.

### Fine motor skills (FMS)

Five tests were conducted to evaluate FMS in accordance with the methodology previously established [9]: (1) *Purdue Pegboard* (Lafayette Instruments, Lafayette, IN, USA) [33–35]. The objective of the task was to insert as many pegs, collars, or washers as possible into holes on a board in a given time. The test comprises four sub-tests (Supplementary Material 2). The number of pieces placed correctly was recorded; (2) *O’Connor Tweezer Dexterity Test* (Lafayette Instruments, Lafayette, IN, USA) [36]; (3) *Grooved Pegboard* (Lafayette Instruments, Lafayette, IN, USA). The time taken to place all the pegs (in seconds) was measured; (4) *Threading task*: participants had to thread six needles with a black thread as quickly as possible, using their dominant hand. The last two needles presented a higher level of difficulty (smaller needle eye). The time taken to thread the first four needles was recorded, as well as the time taken to thread them all [9]; (5) *Water pouring task*: this task consisted of pouring water into five test tubes [7, 9]. The time taken to complete the task (in seconds), the error in volume (in ml), and the water spilled onto the tray (in ml) were measured.

Similar to the calculations made for visual functions, a single metric was obtained for the fine motor skills, the Overall Fine Motor Skills Score (OFMSS). Higher and positive values of OFMSS indicated better performance of the FMS.

### Statistical analysis

Statistical analyses were performed using the software SPSS 28.0 (SPSS Inc., Chicago, IL) and Origin. The effect size is calculated using data from previous similar research [9]. Firstly, the Shapiro-Wilk test was run on all variables (vision and FMS tasks) to assess the distribution of the data sample. The means and standard deviations were calculated for each visual parameter and FMS metric for normally distributed variables, and median and interquartile range for non-parametric variables. To analyze the differences between the three viewing conditions (BB, FDE, and FBE), a repeated-measures design was applied by running an ANOVA test. When the sphericity assumption was violated, the Greenhouse-Geisser criterion was considered instead of Mauchly’s. When normality could not be assumed, a Friedman test for one-way repeated measures analysis of variance by ranks was conducted. To explore the main effects, pairwise comparisons were calculated, applying a Bonferroni adjustment. The relationship between visual function and FMS was first explored with a bivariate correlation analysis. Depending on the assumption of normality, Spearman and Pearson correlation analyses were conducted. Finally, to determine the global impairment, the OVPS and OFMSS were calculated as described above. The significance level was set at *p* < 0.05 for all tests. Multiple-comparison adjustments were implemented in RStudio. Holm-Bonferroni correction was applied to the p-values of global ANOVA/Friedman test within each group of outcome variables (visual and fine motor skills), while correlation analyses corresponding to associations between the OFMSS and multiple visual performance variables were adjusted using the Benjamini-Hochberg false discovery rate procedure.

## Results

An A priori power analysis was performed in G*Power (v3.1.9.7) for a repeated-measures ANOVA (within-subjects). Based on a conservative estimate of the effect size derived from previous data (η²*p* = 0.20; Cohen’s f = 0.31), a total sample size of 24 participants was required to achieve 95% power at α = 0.05.

The variables that were normally distributed were the assembly task on the Purdue pegboard, the needle threading task, and the water pouring time. For these variables, the analysis employed the analysis of variance (ANOVA) test. The remaining variables analyzed across the various visual conditions exhibited a non-normal distribution (either visual or FMS tasks), for which the Friedman test was applied. No statistically significant differences were identified between the biological sexes regarding visual function and the performance of FMS (*p* < 0.05).

### Binocular vision

The results from the visual assessment are summarized in Table 1 and Supplementary Material 2. As the assumptions of normality were not met, the Friedman test was employed to compare the three conditions. The effect size was estimated using Kendall’s W. A significant effect of the Bangerter foil was identified for all visual functions analyzed, with a large effect size observed for each of them (Kendall’s W > 0.6), except for the Randot test, for which a moderate effect size was observed (W = 0.36).

**Table 1:** Medians and interquartile ranges for the visual parameters studied under the three experimental binocular conditions: baseline (BB), wearing Bangerter foil on the dominant eye (FDE), or wearing Bangerter foils on both eyes (FBE). The statistic for the total comparison is shown, along with the p-values and the effect size (W), and the p-values resulting from the pairwise comparisons are also included

		Baseline	FDE	FBE	Statistic (F/χ^2^_(2)_)	Pairwise comparisons p-value
Visual Acuity (VA) (logMAR)	Near	-0.10 (-0.10 – -0.08)	-0.08 (-0.10 – -0.06)	0.00 (-0.04 – 0.06)	χ^2^_(2)_ = 56.467 p < 0.001 W = 0.856	BB-FBE: < 0.001 FBE-FDE: < 0.001
	Distance	-0.10 (-0.10 -0.08)	-0.08 (-0.10 -0.04)	0.06 (0.02 0.15)	χ^2^_(2)_ = 56.407 p < 0.001 W = 0.855	BB-FBE: < 0.001 FBE-FDE: < 0.001
Mean Contrast Sensitivity	Near	122.8 (119.7 – 124.2)	119.8 (114.5 – 124.2)	74.8 (67.4 – 82.4)	χ^2^_(2)_ = 50.646 p < 0.001 W = 0.767	BB-FBE: < 0.001 FBE-FDE: < 0.001
	Distance	110.6 (104.5 – 120.4)	101.0 (86.1 – 109.7)	62.2 (49.3 – 67.8)	χ^2^_(2)_ = 58.970 p < 0.001 W = 0.893	BB-FDE: 0.006 BB-FBE: < 0.001 FBE-FDE: < 0.001
DG Mean		0.0140 (0.0098 – 0.0253)	0.0260 (0.0207 – 0.0530)	0.0793 (0.0532 – 0.0973)	χ^2^_(2)_ = 49.273 p < 0.001 W = 0.747	BB-FDE: 0.003 BB-FBE: < 0.001 FBE-FDE: 0.001
Stereoacuity (arcsec)	OptoTab Distance	23.0 (11.0 – 40.0)	319.0 (131.0 – 479.0)	274.0 (120.0 – 467.5)	χ^2^_(2)_= 46.609 p < 0.001 W = 0.706	BB-FDE: < 0.001 BB-FBE: < 0.001
	Randot	25.0 (20.0 – 30.0)	30.0 (25.0 – 70.0)	40.0 (30.0 – 70.0)	χ^2^_(2)_= 23.890 p < 0.001 W = 0.362	BB-FDE: 0.020 BB-FBE: 0.010
	Frisby	10.0 (5.0 - 20.0)	30.0 (20.0 – 55.0)	30.0 (25.0 – 75.0)	χ^2^_(2)_ = 41.550 p < 0.001 W = 0.630	BB-FDE: < 0.001 BB-FBE: < 0.001

Better results for all the visual parameters studied were obtained in the binocular baseline condition (BB). There were statistically significant differences between the BB results and the foil conditions (FDE and FBE) for all visual functions. To determine which conditions contributed to these differences, pairwise comparisons were performed. A significant difference was found between BB and FBE conditions for all visual functions. In FBE condition, the mean VA distance was worse than VA near, which could be considered a normal value for adults in near (mean VA_FBE_Near_: 0.98 ± 0.13). Similarly, the mean values of stereoacuity obtained from Randot and Frisby stereotests could be considered normal (< 60 arcsec), but not those obtained from the OptoTab distance stereotest. In the same viewing conditions, the mean values from DG and CS exhibited a significant impairment, indicating greater difficulty in discerning objects in low-contrast situations and in the presence of glare. In addition, a significant difference was found between the BB and FDE conditions for distance CS, DG, and stereoacuity.

When comparing the two impairment conditions (FBE-FDE), statistically significant differences were obtained for all the visual functions analyzed (*p* < 0.05), except for the three tests used to measure stereoacuity. In these cases, the worst results were obtained for both eyes wearing the Bangerter foils.

### Fine motor skills

Table 2 shows descriptive statistics for the different metrics used to characterize fine motor skills (FMS). The ‘foil’ factor was significant in most of the manual tasks, exhibiting a moderate effect size, with values of Kendall’s W ranging from 0.17 to 0.33, and partial η² higher than 0.32 for ANOVA, except in the case of water pouring performance time and the subtest Purdue using the non-dominant hand (partial η²=0.098).

**Table 2:** Mean values (± SD) or median (interquartile range) obtained for the manual tasks in the three experimental conditions: baseline (BB), wearing Bangerter foil on the dominant eye (FDE), and wearing Bangerter foil on both eyes (FBE). The statistics for the total comparison, along with the p-value and the effect size (W/η²) resulting from the pairwise comparisons, are also included

Purdue Pegboard		Baseline	FDE	FBE	Statistic (F/X^2^)	Pairwise comparisons *p*-value
	Dominant Hand (no. pegs)	16.0 (14.0–16.5)	15.0 (13.5–16.0)	14.0 (13.5–16.0)	X^2^_(2)_ = 14.463 *p* = 0.007 W = 0.219	BB-FBE: 0.005
Assemblies (no. pegs)	40.73 (± 6.49)	37.52 (± 6.92)	37.30 (± 6.34)	F _(1.631, 52.19)_ = 8.351 *p* = 0.007 η² = 0.322	BB-FBE: < 0.001 BB-FDE: 0.010	
Both Hands (no. pegs)	12.0 (11.0–13.0)	11.0 (10.0–12.0)	10.0 (10.0–11.0)	X^2^_(2)_ = 21.453 *p* < 0.001 W = 0.325	BB-FBE: < 0.001 BB-FDE: 0.017	
Non-Dominant Hand (no. pegs)	14.0 (13.0–15.0)	14.0 (12.0–14.0)	13.0 (12.0–14.0)	X^2^_(2)_ = 6.496 *P* = 0.111 W = 0.098		
O´Connor	Performance time (s)	35.60 (31.24–39.55)	38.46 (34.16–45.55)	39.10 (36.04–45.85)	X^2^_(2)_ = 10.970 *p* = 0.020 W = 0.165	BB-FBE: 0.004
Grooved	Performance time (s)	57.00 (52.86–63.80)	59.70 (53.53–64.70)	59.91 (57.43–70.33)	X^2^_(2)_ = 21.453 *p* < 0.001 W = 0.325	BB-FDE: 0.001
Needle threading	4-needle performance time (s)	18.91 (± 4.53)	24.74 (± 8.39)	26.98 (± 9.18)	F _(1.591, 81.00)_ = 12.167 *p* < 0.001 η² = 0.602	BB-FBE: < 0.001 BB-FDE: < 0.001
	All-needle performance time (s)	30.79 (± 7.79)	39.70 (± 12.28)	46.12 (± 14.12)	F _(1.412, 45.18)_ = 23.404 *p* < 0.001 η² = 0.735	BB-FBE: < 0.001 BB-FDE: < 0.001
Water pouring	Performance time (s)	37.62 (± 8.72)	35.38 (± 7.57)	35.76 (± 8.61)	F _(2, 64)_ = 3.486 *p* = 0,111 η² = 0.098	
	Water spilled (ml)	0.0 (0.0–2.0)	0.0 (0.0–1.0)	1.0 (0.0–2.0)	X^2^_(2)_ = 11.185 *p* = 0.020 W = 0.169	BB-FBE: 0.029
	Error made (ml)	3.0(1.0–8.0)	4.0 (2.0–6.0)	3.0 (1.0–5.0)	X^2^_(2)_ = 0.455 *p* = 0.796 W = 0.007	

The effect size was included in the table using Kendall´s W and partial η², for non-normal and normal distribution respectively

Better results were obtained in BB condition for all the FMS parameters studied: a higher number of pegs inserted in the Purdue Pegboard tests, and less time spent on the different tests (Grooved and O’Connor tests, water pouring, and needle threading). Thus, the Chi-squared or ANOVA statistic revealed significant differences when considering the experimental condition (BB, FDE, FBE) for all the FMS variables analyzed, except for the variable representing the error made in water pouring (*p* = 0.796). However, according to pairwise comparisons, no significant differences were identified in terms of performance time for the water pouring task and the number of pegs inserted on the Purdue Pegboard with the non-dominant hand.

As shown in Table 2, when comparing BB and FBE conditions, significant differences were found in seven of the eleven FMS variables analyzed. When comparing BB and FDE conditions, significant differences for five FMS variables were obtained.

On the other hand, no statistically significant differences were found when comparing the two Bangerter foil conditions (FBE and FDE) for any of the FMS analyzed.

Additionally, the needle test was analyzed to ascertain the impact of needle difficulty. As the variables in question (threading time per needle) are not normally distributed, a Wilcoxon test was employed to ascertain whether there were statistically significant differences between the two sizes of needles. The median and IQR threading time per needle for the initial four needles was 5.44 (4.36–7.16) s, while the median for the final two needles was 6.06 (4.86–7.86) s, and significant differences between the two threading times per needle were observed (Z = -5.522; *p* < 0.001). The time taken to thread one needle increased significantly when decreasing the hole size and, therefore, when increasing the level of difficulty.

### Correlation between binocular Visual performance and FMS

The results of the significant correlations between each visual variable and the manual dexterity tasks under the three viewing conditions have been obtained and shown in Table 3.

**Table 3:** Significant correlations between the needle threading task and visual functions measured at near. Spearman coefficients and p-values were also included

		Visual Acuity Near (VA)	Mean Near Contrast Sensitivity	Disability Glare Mean (DG)
Needle threading	4-needle performance time (s)	rho: −0.408 *p*-value < 0.001	rho: −0.418 *p*-value < 0.001	
	All-needle performance time (s)	rho: −0.446 *p*-value < 0.001	rho: −0.471 *p*-value < 0.001	rho: 0.398 *p*-value < 0.001

Statistically significant correlations were observed between visual functions (VA, CS and DG) assessed and the needle-threading tasks (*p* < 0.05). The strongest correlation was obtained between CS in near and the total time required to thread the six needles. These results showed that a higher level of DG took more time to finish the task.

**Fig. 2:**
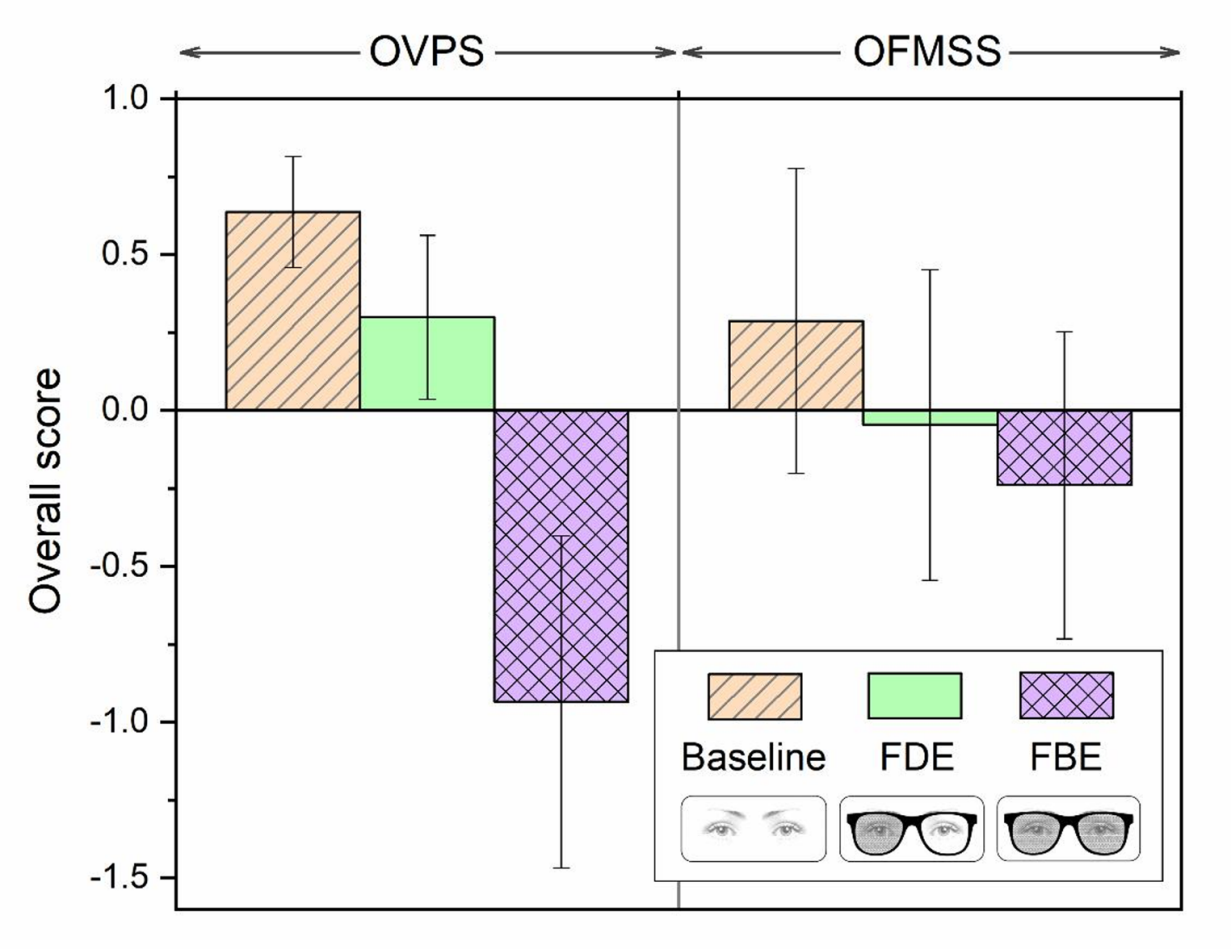
Mean overall scores (OVPS, Overall Visual Performance Score, and OFMSS, Overall Fine Motor Skills Score) in the three binocular viewing conditions: binocular baseline (BB), Bangerter foil on dominant eye (FDE), and Bangerter foil on both eyes (FBE). Standard deviations included

The OVPS for each of the three experimental conditions (BB, FDE, FBE) was obtained. Figure 2 shows how the OVPS decreases as the induced visual impairment increases (in the FDE and, especially, in the FBE).

The results for OFMSS are also shown on the right side of Fig. 2, indicating that the OFMSS decreases as the level of binocular visual impairment increases. In summary, both visual performance and performance of fine motor skills deteriorated with induced visual impairment, in such a way that the greater the induced binocular impairment, the worse the vision and the ability to perform fine motor tasks.

**Fig. 3:**
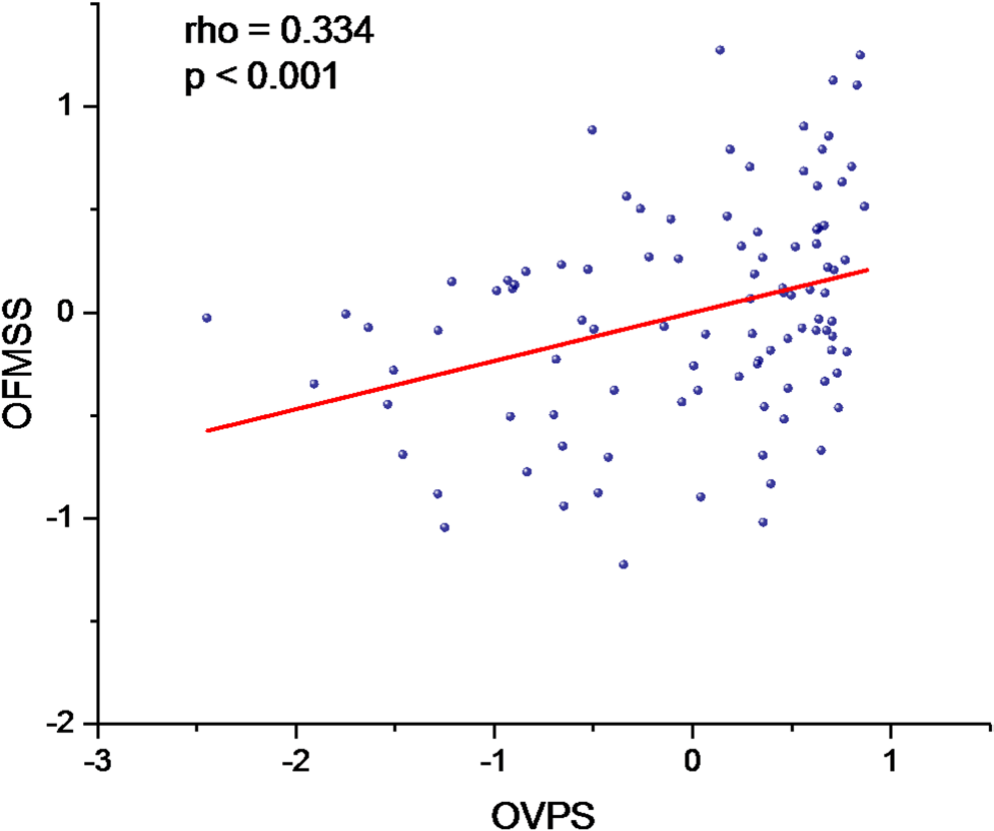
The overall fine motor skills score (OFMSS) as a function of the overall visual performance score (OVPS)

A small-to-moderate, yet significant, positive correlation was found between OVPS and OFMSS (rho = 0.334; *p* < 0.001) (Fig. 3). This correlation indicates that the better the visual functions (VA, CS, DG, and stereoacuity), the better the performance on tasks involving FMS.

Additionally, the relationship between the OFMSS and each visual function was also explored. The findings revealed a significant positive correlation between the OFMSS and each of the following visual functions: near VA (rho: 0.295, p: 0.004); distance VA (rho: 0.273, p: 0.006); mean near CS (rho: 0.360, *p* < 0.001); and mean distance CS (rho: 0.305, p: 0.003). These results show that the better the VA and the CS, the better the ability to perform fine motor tasks. Furthermore, a significant negative correlation was observed between the OFMSS and two additional visual functions: mean DG (rho: -0.366, *p* < 0.001) and stereoacuity (OptoTab distance; rho: -0.433, *p* < 0.001). These findings indicate that impaired stereoacuity and DG (higher values in these visual functions indicate worse visual function) are associated with poorer scores on the FMS.

Finally, to analyze the details of this relationship, the manual tasks that were performed with the use of a standardized test (O´Connor, Groove, and Purdue Pegboard) were separated from the daily tasks that were included in the experiment (needle threading and water pouring). The relationship between FMS (OFMSS) and visual performance (OVPS) was explored for both standardized tasks and daily tasks (supplementary material 3). In both analyses, a significant correlation was obtained between these tasks (OFMSS) and OVPS (rho: 0.257, p: 0.010 and rho: 0.344, *p* < 0.001, for standardized tests and daily tasks, respectively).

## Discussion

First, the results of the visual function tests demonstrated that Bangerter foils impair binocular vision, in accordance with previous studies [5, 15, 27]. This is due to the structure of the foil, which produces a higher amount of intraocular straylight, resulting in a poorer-quality image on the retina [15]. The deterioration obtained in VA, CS, DG, and stereopsis is greater when wearing the foils in both eyes (FBE). In these cases, a relevant deterioration in all the visual functions was obtained. Bangerter foils grade 0.8 induce a mild deterioration in retinal image quality and an impairment in monocular visual performance [15, 25, 27, 37]. These foils induce a level of intraocular scattering similar to that obtained in an incipient cataract and a visual impairment such as that of edema and corneal dystrophies [5, 15, 19, 38, 39].

FDE revealed deficits in binocular visual performance, particularly in terms of distance CS, DG, and stereoacuity. However, the outcomes for VA and near CS in natural binocular vision (BB) of participants, and in FDE, are strikingly similar [5]. The FDE results may be of particular relevance to individuals experiencing unilateral vision loss. It appears that these individuals do not exhibit a heightened prevalence of problems with their binocular VA. However, it has been observed that the presence of glare (which has been shown to induce a greater degree of impairment) can serve as a significant contributing factor to the deterioration of visual performance [14, 15]. This mild visual impairment (affecting only one eye) affects stereopsis more than VA and CS in binocular viewing conditions. This may be because binocular summation compensates for visual impairment in the case of binocular VA and CS [5, 15, 27]. In the case of stereopsis, a discrepancy was observed between the three tests employed. This finding might be attributable to the inherent properties of the measurement methods employed. This impairment was more relevant when assessing true depth in near vision (Frisby) compared to the test in dissociated vision conditions, although stereopsis was also strongly affected in far vision. Our results from monocular or binocular impairment were similar and could be considered as normal values (stereoacuity < 40 arcsec). Furthermore, the findings exhibited discrepancies when compared to previous studies [5, 40]. Monocular VA degradation appears to have a mild but more significant effect on stereoacuity than binocular degradation in moderately reduced VA ranges (0.8 to 0.4) [40].

Martino et al., (2023) demonstrated that stereoacuity at distance is correlated with interocular differences (ID), indicating a deterioration of stereopsis with increasing ID [16], depending mainly on the level of scattering [15, 41]. However, it is also possible that stereoacuity could be worse in FBE because both monocular images deteriorate, thus affecting binocularly, although the ID are smaller. In the case of FDE, deterioration of vision occurs bilaterally due to monocular impairment, except for binocular near VA and CS. These findings are consistent with previous studies that used other types of visual impairment, such as defocus, with positive lenses [31]. Bangerter foils increase intraocular straylight, reducing CS and affecting VA, obtaining results comparable to those obtained for different degrees of ocular pathologies (e.g. cataract), depending on the type of Bangerter foils used [5, 27, 37]. However, the use of positive lenses produces a defocus in the retinal image, modifies the accommodative capacity and affects VA, but does not introduce the straylight similar to ocular pathologies such as cataracts.

Furthermore, visual conditions characterized by diminished contrast and reduced stereoacuity have been demonstrated to exert a substantial impact on visual function [5, 7, 15, 16, 19]. Consequently, these factors may potentially influence the performance of daily tasks that demand the estimation of distances.

That binocular vision surpasses monocular vision in terms of manual dexterity has been supported by numerous studies [7, 9, 21, 42]. In the same line, our results demonstrated a strong impairment in FMS in FBE and FDE (seven and five of the eleven analyzed parameters of the FMS deteriorated significantly), respectively. This means that even if one eye had good visual quality, interocular differences impaired binocular vision and FMS. The five manual tasks assessed in the present study require different visual demands and motor agility [5, 7, 33–36]. In the peg insertion tasks of standardized tests (Purdue, O’Connor and Grooved), significant differences were obtained between visual impairment conditions (FBE and FDE) and baseline condition; however, the number of pegs inserted, and performance times obtained in these three standardized tests showed a smaller range of differences compared to the rest of the tests (threading and water pouring task). This discrepancy could be attributed to the presence of a significant spatial reference, namely the boards themselves, which might facilitate task performance. Furthermore, in the assembly task (Purdue 4-sub-test), the integration of both hands and the increased level of complexity might have contributed to the larger ranges of differences observed between baseline and the two impaired viewing conditions. This could also explain the fact that an increased difficulty in the O’Connor test, where tweezers were used in the peg insertion, led to poorer performance in the filter conditions compared to that observed when using hands (Purdue and Grooved). As was evidenced in preceding studies, degraded binocular functions exhibited varied responses in relation to task difficulty [43]. In the context of needle threading, the complexity of the task demands a finer stereopsis (eyelet and thread exhibit remarkably fine details). This hypothesis is substantiated by the outcomes, demonstrating an increase in the time taken to complete the task when using the two needles that presented more substantial challenges.

The present study has shown significant correlations between each visual function, in terms VA, CS, DG, and stereopsis, and the global index of the FMS calculated (OFMSS). The correlations obtained were small-to-moderate, with the different visual functions explaining approximately 8% to 19% of the variability of fine motor skills. These findings note that, in our opinion, it seems likely that impaired visual functions contribute causally to worse performance on FMS. Considering the OVPS, worse values were observed for the FBE condition compared to the FDE condition. The OFMSS yielded similar outcomes. This would suggest the presence of a potential correlation between the two variables. Indeed, the better the visual performance was obtained, the better the ability to perform FMS.

A previous study revealed that simulated monocular blur had less impact on horizontal tracking than binocular blur, yet it was as detrimental to tracking in depth as binocular blur [44]. It should be noted that, in our study, the tasks performed required the use of both horizontal and depth eye movements, which may have contributed to the lower OFMSS observed compared to the differences obtained in OVPS between each impairment binocular viewing condition.

Comparing the association between vision and the two types of manual dexterity tasks, i.e., daily tasks (water pouring and needle threading) and standardized tests (O’Connor, Purdue, and Grooved), it can be stated that vision has a greater weight in the daily tasks selected in the present study. This finding may be attributed to the spatial references and the varying difficulty between tasks, as previously discussed, and the important role of depth perception for threading and pouring without contact and sensitive feedback. However, factors other than vision may be involved due to the repetitive nature of the standardized tasks. Attention and eye movements could also explain some of the results obtained in the FMS tasks. Thus, it would be interesting to track eye movements to control fixation and to monitor hand movements in terms of velocity and precision under different viewing conditions to provide further information on the performance of FMS, as other authors have done [45]. These issues will be addressed in future research.

These results may shed light on the possible limitations that patients with a low degree of unilateral visual impairment (due to cataracts, corneal edema, or emmetropization processes as monovision) may face in their daily lives; knowing that the simulations do not fully replicate the optical or neural effects of true pathology.

## Conclusions

This work demonstrated that visual performance, in terms of VA, CS, DG, and stereopsis, is affected by either monocular or binocular impairment. Binocular impairment is stronger than monocular impairment, except for stereopsis, which deteriorated similarly in both conditions. This could indicate that even a relatively minor monocular or binocular impairment, possibly due to lower amblyopia or minor ocular media transparency problems (such as an incipient cataract), can have a significant impact on the overall visual system. Visual impairment has been demonstrated to have a detrimental effect on the performance of fine motor tasks. Therefore, in cases of not very high interocular differences, the performance of manual dexterity tasks is affected. It would be interesting to study whether this impairment in FMS could have a negative impact on the lives of these patients. In light of the differences observed between the manual tasks analyzed (in terms of visual demand and motor agility), it is crucial to conduct further investigations in this area. That information could be useful not only in optometry and ophthalmology, but also in the fields of kinesiology, neurology, and psychology.

## Supplementary Information

Below is the link to the electronic supplementary material.

Supplementary figure 1 (2.57 MB)

High Resolution Image (TIF 9.53 MB)

Supplementary figure 2 (327 KB)

High Resolution Image (TIF 1.85 MB)

Supplementary figure 3 (613 KB)

High Resolution Image (TIF 1.85 MB)

## Data Availability

The datasets generated and/or analysed during the current study are available from the corresponding author on reasonable request.
