# Supplementary figures and images for: Impaired binocular vision with bangerter foil and its influence on fine motor skills: a clinical observational, randomized and cross-sectional study
Source: Graefes Arch Clin Exp Ophthalmol. 2026 Mar 23;264(7):2129–40. doi: 10.1007/s00417-026-07150-2 (PMC13341970; doi:10.1007/s00417-026-07150-2)

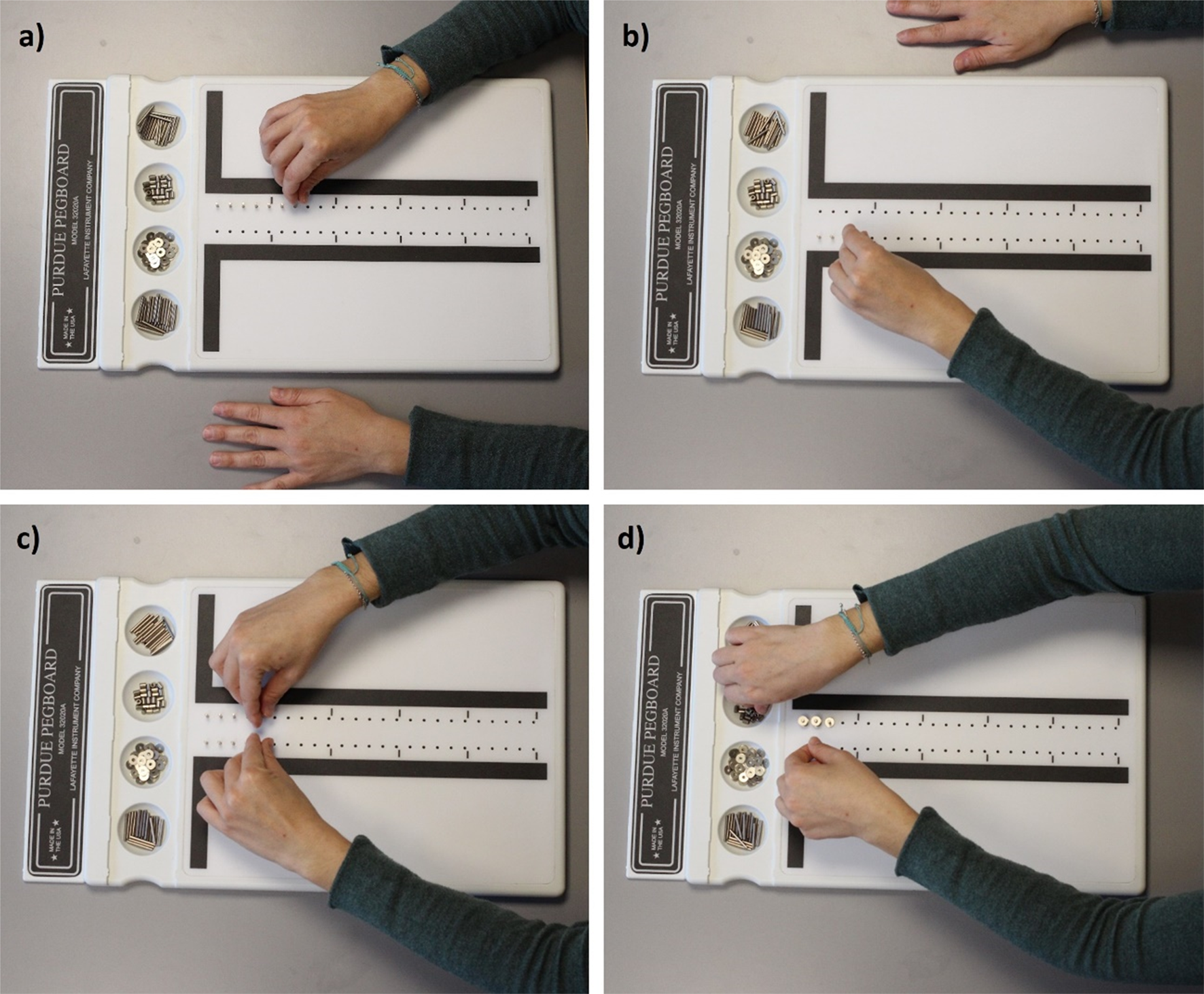

Supplement: Supplementary file 1 — (2.57 MB) [file 417_2026_7150_Fig4_ESM.png]

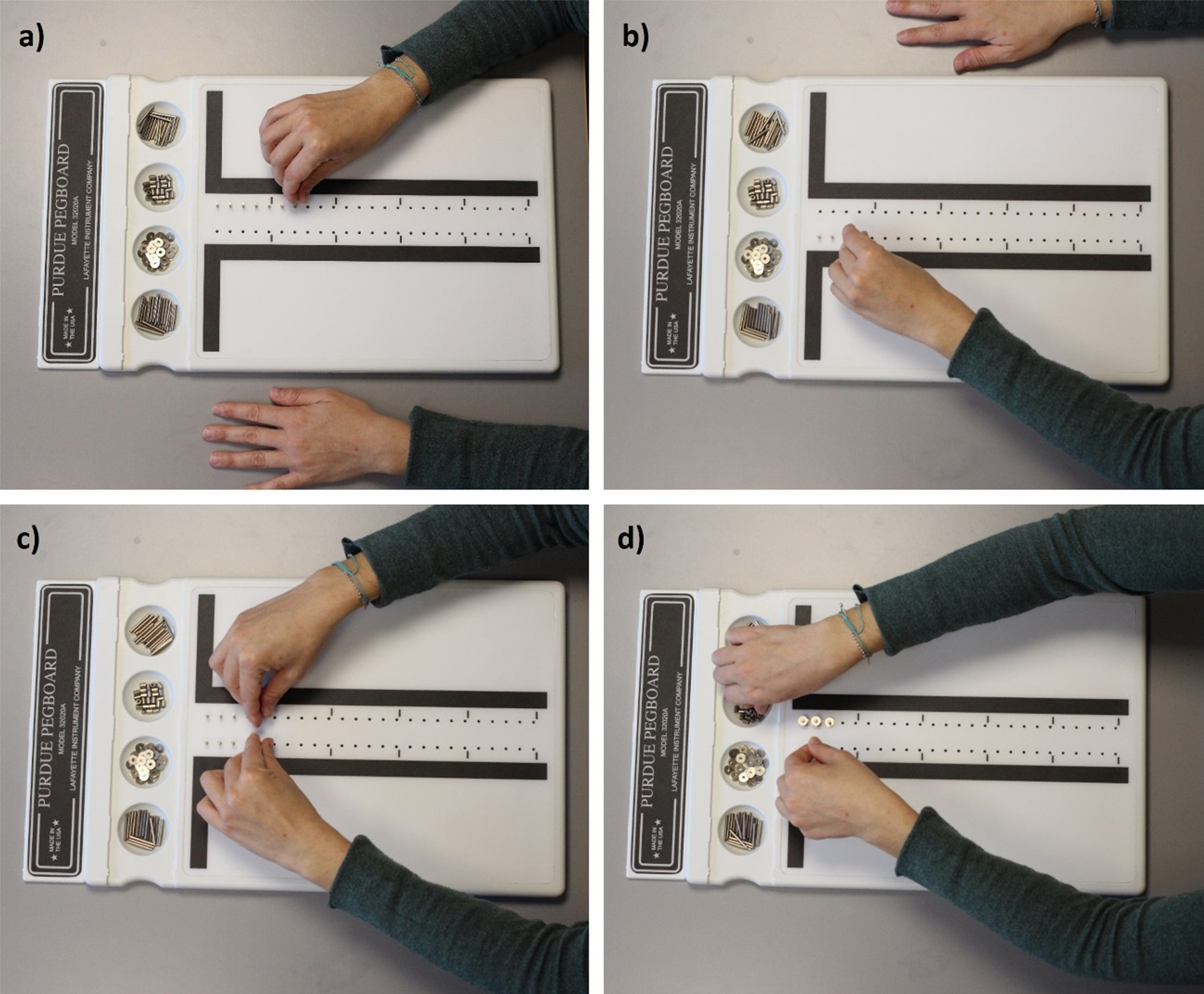

Supplement: Supplementary file 2 — High Resolution Image (TIF 9.53 MB) [file 417_2026_7150_MOESM1_ESM.tiff]

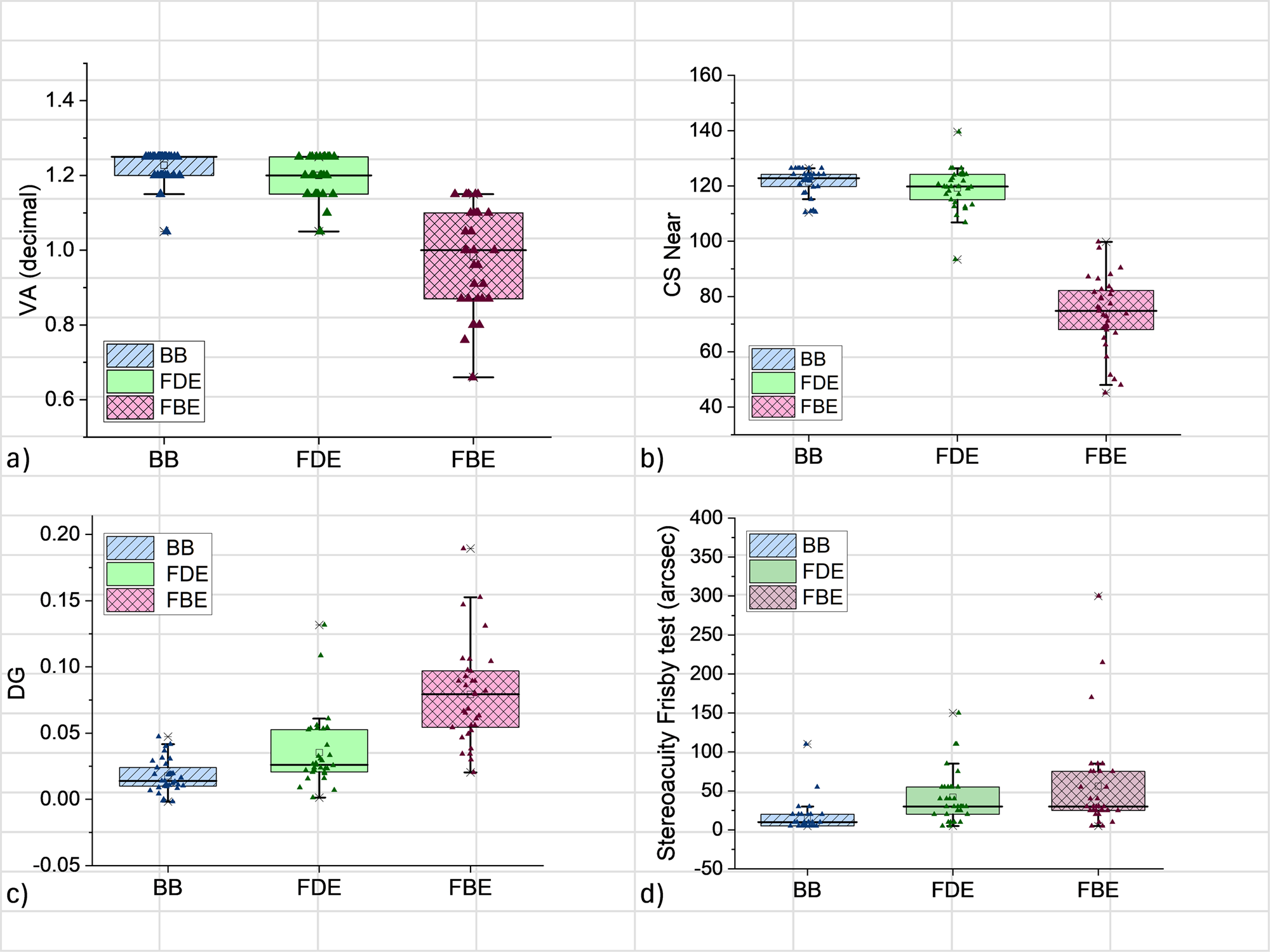

Supplement: Supplementary file 3 — (327 KB) [file 417_2026_7150_Fig5_ESM.png]

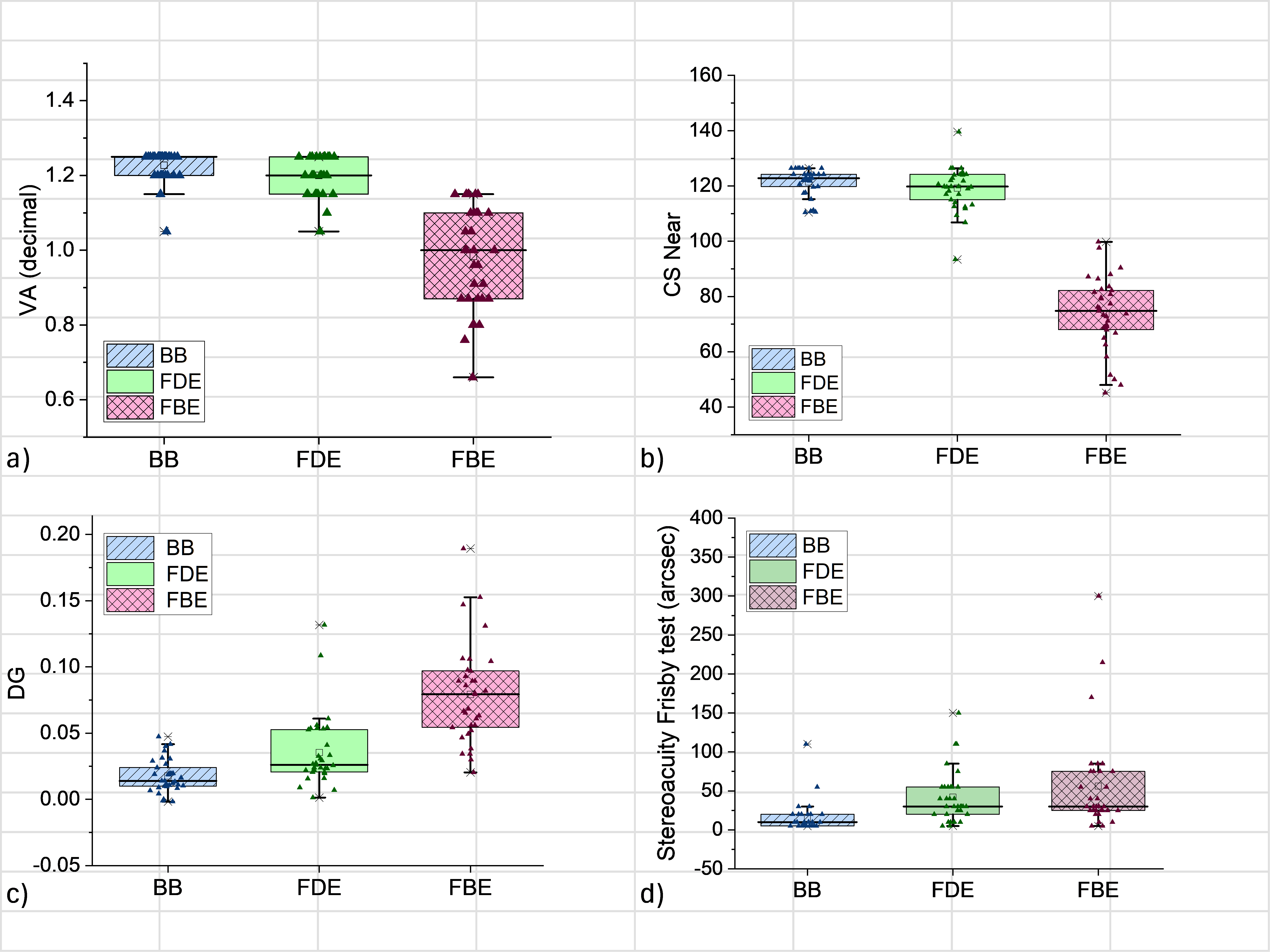

Supplement: Supplementary file 4 — High Resolution Image (TIF 1.85 MB) [file 417_2026_7150_MOESM2_ESM.tiff]

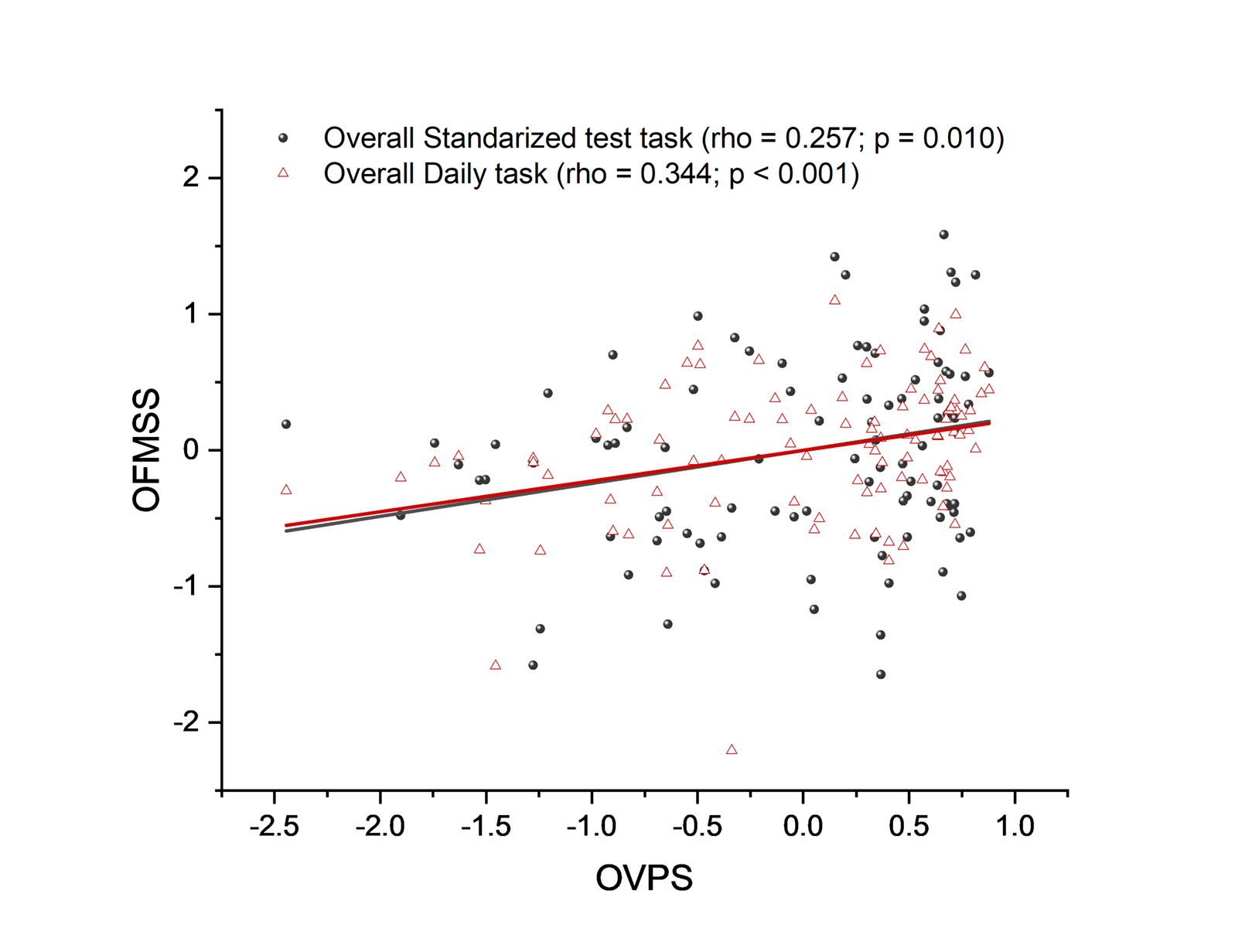

Supplement: Supplementary file 5 — (613 KB) [file 417_2026_7150_Fig6_ESM.png]

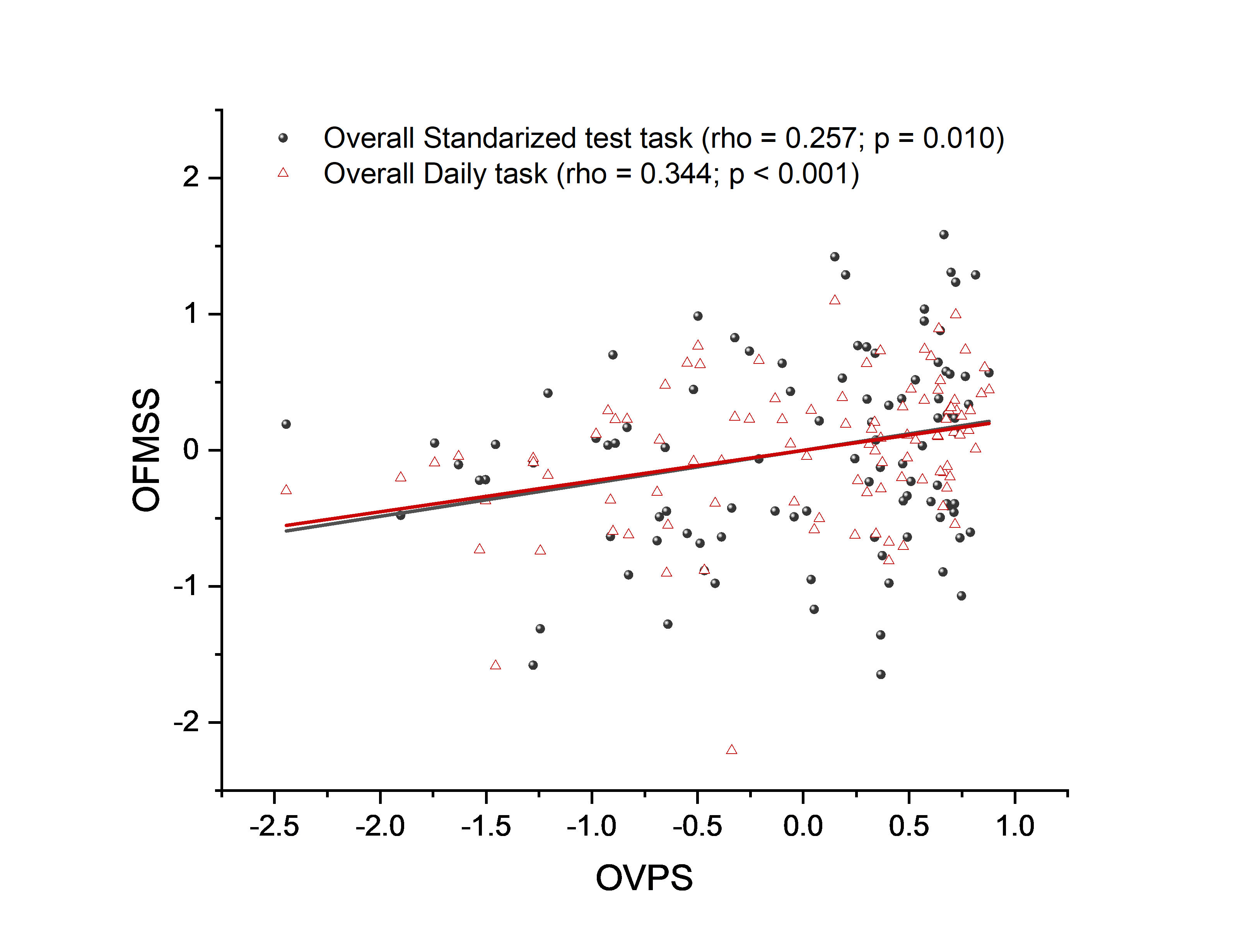

Supplement: Supplementary file 6 — High Resolution Image (TIF 1.85 MB) [file 417_2026_7150_MOESM3_ESM.tiff]
